# Species traits and connectivity constrain stochastic community re-assembly

**DOI:** 10.1038/s41598-017-14774-2

**Published:** 2017-10-31

**Authors:** Rebecca E. Holt, Christopher J. Brown, Thomas A. Schlacher, Fran Sheldon, Stephen R. Balcombe, Rod M. Connolly

**Affiliations:** 1 Australian Rivers Institute, School of Environment, Griffith University, Gold Coast, Qld, 4222 Australia; 2 Centre for Ecological and Evolutionary Synthesis (CEES), Department of Biosciences, University of Oslo, Blindern, 0316 Oslo, Norway; 3 Australian Rivers Institute, Griffith University, Nathan, Qld, 4111 Australia; 4 School of Science and Engineering, The University of the Sunshine Coast, Maroochydore, Qld 4558 Australia; 5 Australian Rivers Institute, School of Environment, Griffith University, Nathan, Qld, 4111 Australia

## Abstract

All communities may re-assemble after disturbance. Predictions for re-assembly outcomes are, however, rare. Here we model how fish communities in an extremely variable Australian desert river re-assemble following episodic floods and drying. We apply information entropy to quantify variability in re-assembly and the dichotomy between stochastic and deterministic community states. Species traits were the prime driver of community state: poor oxygen tolerance, low dispersal ability, and high fecundity constrain variation in re-assembly, shifting assemblages towards more stochastic states. In contrast, greater connectivity, while less influential than the measured traits, results in more deterministic states. Ecology has long recognised both the stochastic nature of some re-assembly trajectories and the role of evolutionary and bio-geographic processes. Our models explicitly test the addition of species traits and landscape linkages to improve predictions of community re-assembly, and will be useful in a range of different ecosystems.

## Introduction

Patterns of community re-assembly following disturbance events can be critical in shaping the function of ecosystems^1–5^. Variability in community re-assembly is often overlooked, despite it being recognised as a promoter of diversity^6^. Stochastic processes in particular can be important in resetting the trajectory of assemblage recovery after major disturbance events^7–9^. For example, neutral theory suggests that in many natural systems patterns of diversity and abundance may be recreated as a result of stochastic processes, of, colonization and extinctions^10,11^. Conversely, deterministic theories of community ecology suggest that local, niche-based processes interact with environmental conditions as well as interspecific interactions, such as mutualism, predation parasitism, and competition, to determine patterns of species diversity and community assembly^12^. Connectivity among habitats at various spatial scales may also influence community re-assembly, by facilitating or restricting the movement of individuals across land- and seascapes^13^, thus influencing species quantity and colonisation.

The processes of both colonisation and extinction can be dependent on evolutionary differences in life history traits^14^ that govern species persistence. Therefore, community re-assembly can also be shaped by species traits^14–17^. Traits that influence species fitness and survival, along the environmental gradient of interest, will be most important for the processes of community re-assembly^18^. The importance of traits is reflected in the utility that trait-based approaches have in predicting community structure in both terrestrial and aquatic environments^19,20^. For example, life-history traits predict use of ephemeral water-bodies versus main channels by Amazonian fishes^21^, and the traits of freshwater fishes across the Australian continent vary consistently with climate-hydrological landscape gradients^22^; and species traits can facilitate plant community assembly in both early and late stages of assembly^15,16,23–25^. Community re-assembly following disturbance can thus be thought of as a sorting process, filtering particular species according to their traits^18^.

Ephemeral rivers are considered to be the most hydrologically dynamic of all freshwater ecosystems^26–28^. These habitats experience episodic flood events and extended periods of drought that can leave entire sub-catchments dry^28–30^. In these systems, fish communities re-assemble during and after periodic flood events that allow fish to migrate along connected channels between discrete waterbodies. In between flood events the channels initially dry. If time increases between flows, water bodies also gradually dry-up and species are lost. We define community re-assembly as the predictability with which a similar assemblage of species will re-assemble at a site (in our case a waterhole) after a disturbance event (in our case a flood). Freshwater fish communities possess a diverse range of species traits; often occupying dynamic habitats with broad ranges of connectivity. Hence, here we use freshwater fish communities in the arid interior of Australia inhabiting ephemeral rivers and waterholes as a case study; to quantify the stochasticity of community re-assembly in freshwater fish communities in ephemeral rivers.

We measured variability in community state using information entropy. Information entropy is widely used to measure variation, or uncertainty, in the outcome of ecological and non-ecological assembly processes^31^. It has, for example, been used in species distribution models^32,33^, to quantify species diversity^34^ (Shannon’s information entropy^35,36^) and to predict the role of environmental variability in determining biodiversity (Shipley’s maximum entropy;^5,15^). However, it has not been applied to study the processes and dynamics (e.g. environmental and ecological) of community re-assembly following disturbance events. Here, we use Shannon information entropy, a novel approach, to quantify uncertainty in community state in ephemeral river systems. High entropy systems have the ability to evolve toward a different stable regime with a new characteristic structure^37,38^. When entropy is low, the converse is true; the system becomes more predictable and re-assembly to a given state will be more deterministic. Low entropy systems may have higher resilience to disturbances, because they tend to re-assemble in the same way time and time again^9^. Our focus on community state and re-assembly is similar to that of Holling’s model of the adaptive renewal cycle. The adaptive cycle model focuses upon the processes of destruction and reorganisation providing a more complete view of system dynamics, organisation and resilience^39^.

We use information entropy to quantify uncertainty in community re-assembly processes, measuring the limits of known deterministic controls on re-assembly within a community assembly model. Predicting variation in the community assemblage based on species traits and connectivity are key issues in community ecology because they inform how ecosystems are structured and function. We specifically investigate how community re-assembly is shaped by two potentially important drivers, individual species traits and connectivity.

## Results

**Figure 1:**
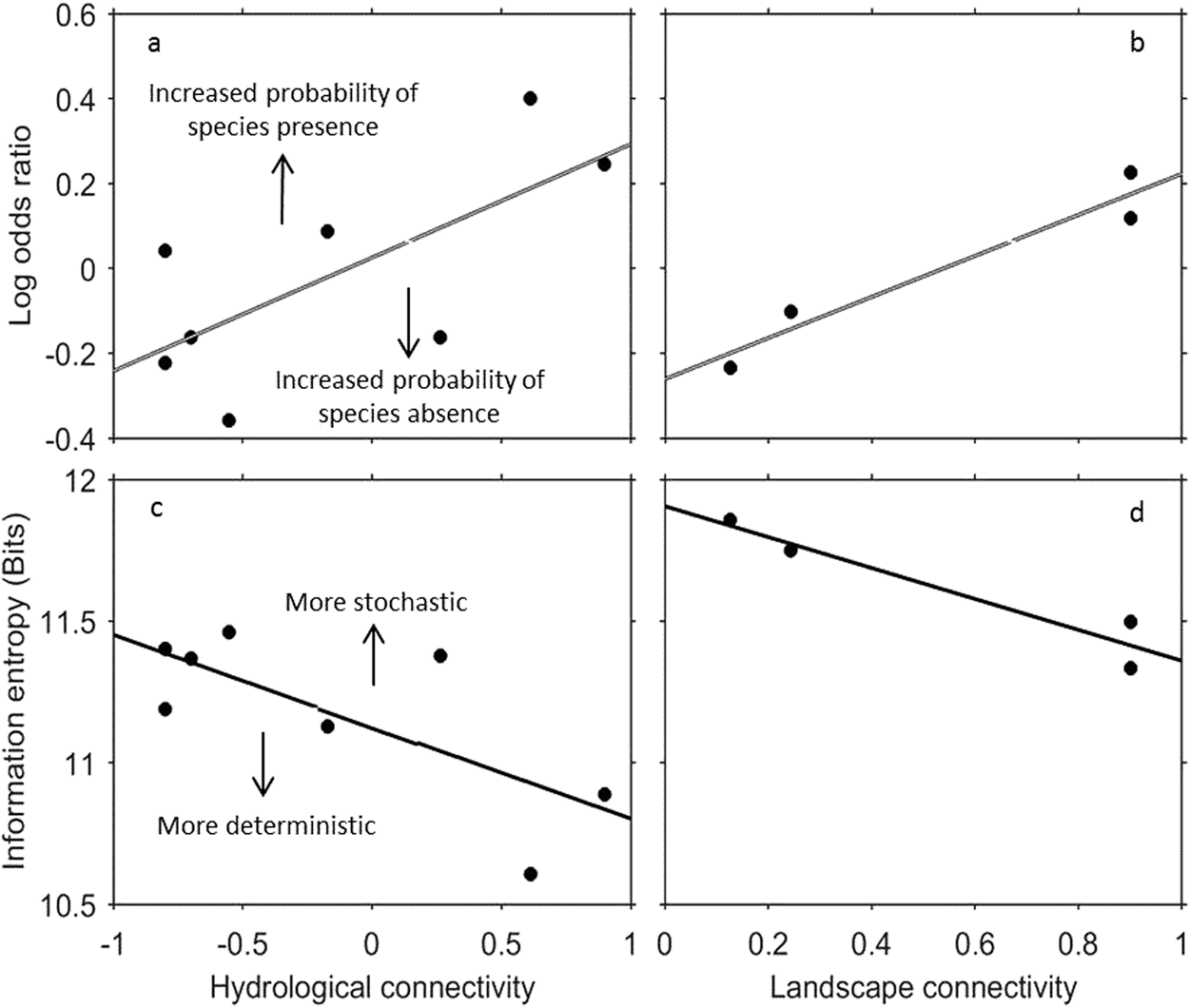
(**a**) Linear regression for log odds ratio (effect of sampling date) and standardised hydrological connectivity, *R* ^2^ = 0.49, *P* = 0.05. (**b**) Linear regression for odds ratio (effect of waterhole) and landscape connectivity, *R* ^2^ = 0.90, *P* = 0.05. (**c**) Linear regression for community entropy (mean for all waterholes) and standardised hydrological connectivity, *R* ^2^ = 0.53, *P* = 0.04. (**d**) Linear regression for community entropy (mean for all waterholes) and landscape connectivity, *R* ^2^ = 0.83, *P* = 0.09.

The three species traits that were most important in predicting the probability of species occurrence were: oxygen tolerance, dispersal ability, and total fecundity (Table 1). In addition to these species traits, the degree to which assemblages were connected (in space and time) determined the probability of species presence. As hydrological connectivity increased, so did the probability of species presence, indicated by a positive relationship between the log odds ratio from the random effect ‘sampling time’ and hydrological connectivity (Fig. 1a). Landscape connectivity also had a positive relationship with the log odds ratio, for the effect of waterhole (Fig. 1b). As landscape connectivity decreased (i.e. distance among waterholes increased), the probability of species absences increased, suggesting that less-connected waterholes are more likely to have fewer species.

**Table 1:** Summary of significant fixed effects terms in the generalised linear mixed-effects models, testing how species traits predict species presence and absence (S.E: Standard Error; Z: Z statistic; *P*: significance level).

Traits	Value	S.E	*Z*	*P*
Medium oxygen tolerance	1.113	0.426	2.609	0.009
High oxygen tolerance	2.174	0.318	6.819	<0.001
Low fecundity	1.051	0.350	2.997	0.002
Medium dispersal ability	1.313	0.459	2.862	0.004
High dispersal ability	1.832	0.350	5.229	<0.001

As hydrological connectivity increased, during times when waterholes were less isolated, information entropy decreased, indicating that community re-assembly became more deterministic (Fig. 1c). A similar relationship was found also for landscape connectivity (Fig. 1d). When waterholes were more connected, information entropy was low and the community state became more deterministic (this relationship had a high *R* ^2^ but was not significant at *P* = 0.05, Fig. 1d; *R* ^2^ = 0.83, *P* = 0.09).

**Figure 2:**
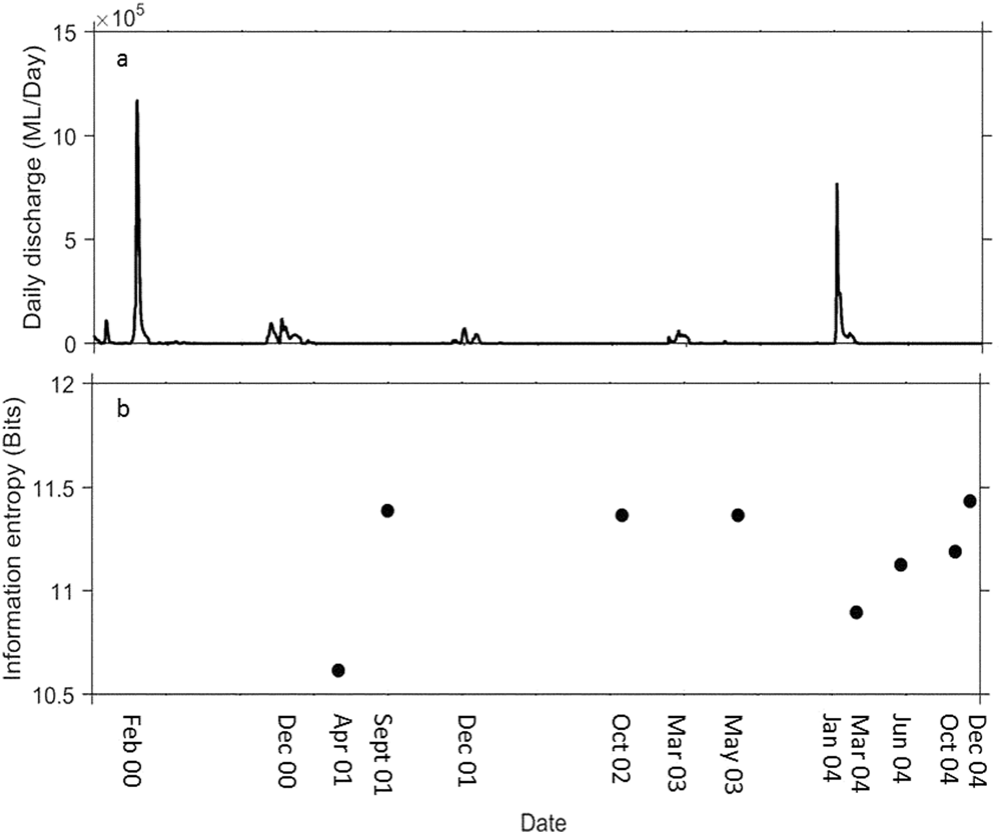
(**a**) Daily discharge of the Cooper Creek (derived from the sum of discharge in the Thomson and Barcoo Rivers) from February 2000 to December 2004 (Bureau of Meteorology). Arrows indicate fish sampling events. (**b**) Information entropy results (for all waterholes), for each sampling date.

Entropy was lowest for assemblage data collected during the first sampling event after floods in 2001 and 2004 (Fig. 2b). At these times, the probability of all species within the sampled assemblage being present was high and the potential variability in community state was low. As the river network dried up during a longer period of drought, the assemblage variability shifted towards a more stochastic state, illustrated by increasing information entropy values following the March 2004 flood event (Fig. 2b).

**Figure 3:**
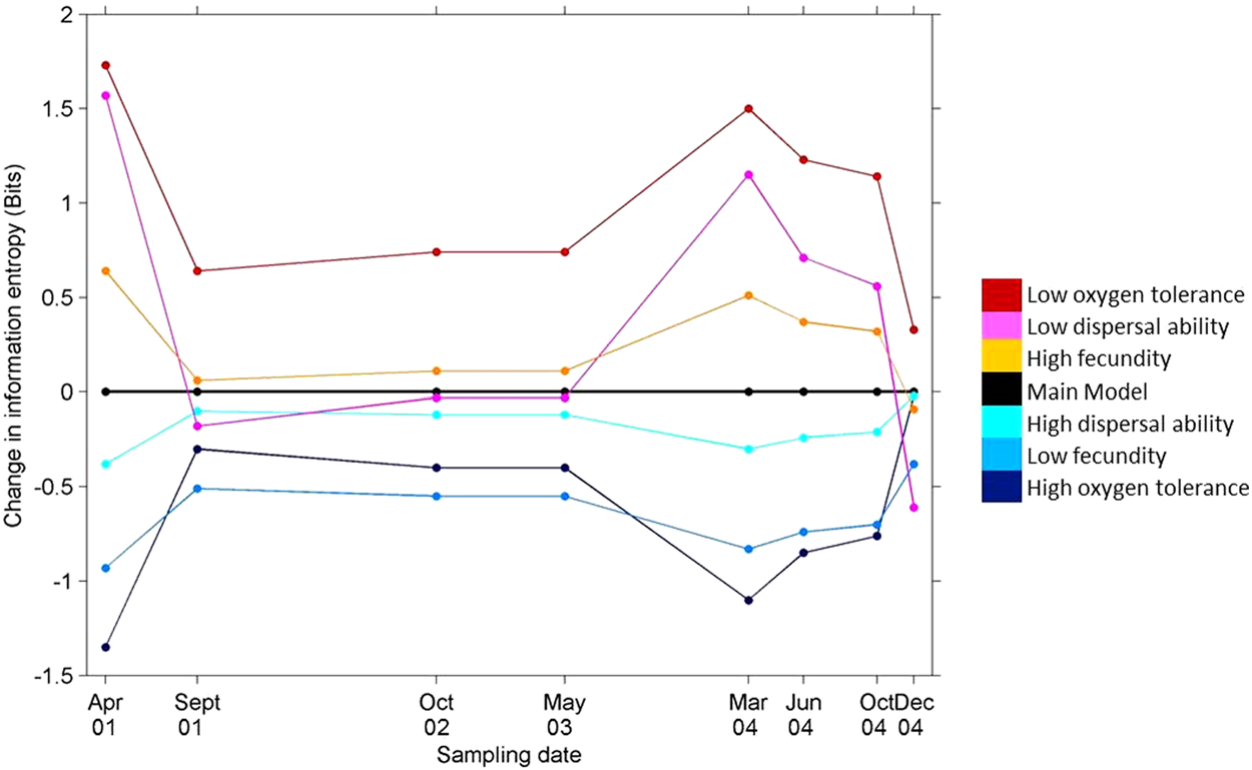
Community entropy (mean for all waterholes), for each sampling date, following trait filtering. Coloured circles represent different trait manipulations as per legend; black represents the main model, no filtering.

Trait filtering had variable effects on community entropy (Fig. 3). Entropy increased at all sampling times if: a higher proportion of species possessed high oxygen tolerance (Fig. 3 - dark blue), low fecundity (Fig. 3 - blue) or high dispersal ability (Fig. 3 - cyan) traits (Fig. 3). Entropy decreased if a higher proportion of species possessed traits consisting of low oxygen tolerance (Fig. 3 - red), low dispersal ability (Fig. 3 - magenta) and high fecundity (Fig. 3 - orange). Trait filtering on the oxygen tolerance traits (Fig. 3 - red and dark blue), represented the largest change in community entropy.

## Discussion

We found that individual species traits drove variation in community re-assembly of a river fish assemblage. Higher connectivity also reduced variability in reassembly, however to a lesser extent than did traits.

Species traits relating to oxygen tolerance, fecundity, and dispersal ability were found to be important for the probability of species presence, community re-assembly, and information entropy within our study. The importance of these traits is not surprising. Due to the dynamic nature of ephemeral rivers, dissolved oxygen concentrations vary considerably^27^; species that are physiologically adapted to low oxygen environments are thus more likely to be present and persist following disturbance events. The maturation of freshwater fishes in ephemeral rivers is often synchronised with the flood regime, which ensures that suitable post-spawning conditions are present, and recruitment is high^30,40,41^. In our study, low fecundity in particular, was found to shift communities toward a more deterministic state, whereas the opposite trend was predicted when high fecundity traits were filtered to be more prevalent. Life-history adaptations to flooding and drought are facilitated by trade-offs with survival^42^. By synchronising maturation and reproductive events to the timing of flood seasons, adult mortality is reduced and survivorship of young increased^42^.

It should be noted that our modelling approach predicts community re-assembly but does not take into account species interactions within that community. Species interactions may be incorporated in the future, by using methods such as MARS (Multivariate adaptive regression spline) models. MARS provide an alternative method for fitting non-linear regression responses that utilise precise linear fits rather than smoothing functions as used in GAMS (Generalised additive model)^43,44^. For this particular system, however, inter-specific interactions are likely of similar magnitude to intra-specific interactions because all species occupy similar trophic niches^30,45^. Hence the Cooper Creek ephemeral river represents a suitable case study to develop our modelling methodology without hindering predictions.

Connectivity was also found to have an effect on information entropy and the re-assembly of communities, although to a lesser extent than species traits. Our model predicts that when waterholes are better connected following significant river flow, the assemblages become less stochastic. The most plausible mechanism for more deterministic assemblages during these times is enhanced movement and exchange of individuals across the stream network with fish able to make multi-directional movements (longitudinal and lateral) toward favoured habitats within these systems^41^.

Flood events in the lowlands of floodplain rivers allow unhindered movement of individuals across the floodplain network, resulting in species retention and gain within waterhole refugia^45^. As such, connectivity is a key factor in freshwater systems^46^. Highly vagile macroinvertebrates, including facultative aquatic insects such as beetles and hemipterans, may be less constrained by dispersal ability with the capacity to disperse through the terrestrial environment. Conversely, obligate aquatic organisms such as fishes and amphibians are bound to the geography and hydrology of these systems^46^. The degree of connectivity will thus influence community structuring and may change over time^46^. In our study, hydrological connectivity changed over the sampling period due to episodic floods and periods of drought. As such, information entropy response after disturbance events was diverse (Fig. 3); communities shifted towards different variants of deterministic-stochastic community states depending on the sampling date (Fig. 3). In the Pantanal floodplain system in South America, seasonal patterns of metacommunity structure were found^46^. During the flood season, community structuring was spatially nested and species co-occurrence important, whereas at the end of the wet season, environmental factors were found to be more important^46^.

Species dispersal ability is also suggested to influence community re-assembly and information entropy. It is generally accepted that community reorganisation following a disturbance event is driven by dispersal and niche-based mechanisms^14,47,48^. Dispersal ability or the level of connectivity of habitat provides opportunities for adaptation via dispersal, maintaining habitat resilience^49^. In the upper Paraná River floodplain (Brazil), the dispersal ability of several different groups of organisms including both migratory and non-migratory fish determined the relative role of environmental and spatial processes on structuring local communities^50^. In ephemeral rivers where spatial patterns of connection can be variable and environmental influences on survival strong^30,51^ the ability for individual species to disperse and move to other parts of the ecosystem is thus fundamental to their long-term survival and persistence^27,52^.

Data used in our study were concentrated within a cluster of four waterholes on the Windorah reach of the Cooper Creek system and as such there was a limited gradient in connectivity metrics. This may have resulted in the weak effect of landscape connectivity on entropy, which may be stronger in more spatially heterogeneous systems. Our study provides a novel example of how information entropy can be utilised to quantify variability in community re-assembly within disturbed habitats that could be applied to more temporally and spatially extensive datasets.

## Conclusions

There are limits in the predictability of community re-assembly following disturbance. Our modelling approach enables predictions for the likely state of the assemblage across a spectrum from stochastic to deterministic states. It can also test how permutations of species trait expressions in conjunction with connectivity measures (both in space and time) shape the variance in assemblage uncertainty.

Our use of information entropy in this context is valuable; presenting a flexible modelling approach that can be developed further to answer questions relating to ecosystem function and can be readily applied not only to other freshwater systems but equally in a range of marine and terrestrial environments.

## Methods

### Study region

We analysed community re-assembly in fish from a number of waterholes in creeks within the Cooper Creek catchment in Queensland, Australia. The Cooper Creek catchment has a semi-arid climate, with mean rainfall ranging from <100 to 500 mm annually^51^. The majority of stream flow is generated by monsoonal rain in the headwaters, and intermittent local rainfall, resulting in highly variable flood pulses, characterised by boom and bust periods of flooding alternating with droughts^30^. During droughts, which can last several months or longer^29^, the waterholes become disconnected from one another and from the main floodplain; individual waterholes that remain with water then serve as refugia for fish^53^. Conversely, episodic floods can inundate the floodplain and reconnect channels, tributaries, and waterholes that became isolated during the drought^41^.

For this study we complement a published dataset (data were originally collected and made available by Arthington *et al*.^51^, Balcombe and Arthington^30^ (Fish presence/absence for each time and waterhole; Table S1), with newly collected data on hydrological connectivity and species traits, assembled specifically for the purposes of our study. Fish were sampled on eight occasions (April and September 2001, October 2002, May 2003, March, June, October and December 2004; see Arthington *et al*.^51^, Balcombe and Arthington,^30^) (Fig. 2a, Table S1) in four waterholes of the Windorah reach of Cooper Creek, within the Lake Eyre Basin, central Australia (S 25°428, E 142°734). During the April 2001 sampling trip, several physical variables were measured (see supplementary material for those used in analyses, Table S2). Fish were sampled using a standard protocol of three double-winged fyke nets (standardised for wing width and soak time) and one beach seine (standardised to a 10 m^2^ benthic area) (See Arthington *et al*.^51^, Balcombe and Arthington^30^ for detailed sampling methods and maps detailing study area).

During the study period there were two major flood events, the first in February 2000 (14 months prior to our first sampling occasion) and the second in January 2004 (2 months prior to our fifth sampling occasion) (Bureau of Meteorology; Fig. 2a). These two events inundated extensive areas of the floodplain, including all channels and waterholes^41^. There were also a number of smaller in-channel flows which occurred in December 2000, 2001, and March 2003 (Fig. 2a). These smaller flows connected all waterholes through channel networks in the Windorah reach. Apart from the flow and flood events described above, individual waterholes were isolated due to zero flows at all other times, drying at a rapid rate

### Species traits

Information was collated for 16 traits for all freshwater fish species sampled (Table S3). Traits describe life-history characteristics, dispersal ability, and tolerance to environmental variables (Table S3). Trait assignments were based on a number of sources of information from the literature, including species accounts in comprehensive texts^25,54–56^. We calculated the median value when only ranges were reported or available; ordinal data were assigned a single trait state (for example, dispersal ability is coded as 1,2,3, with 1 being low dispersal ability and 3 high dispersal ability). If several values were reported for a trait, the mean was calculated.

### Connectivity

To test whether connectivity mediates variability in community re-assembly, two metrics were used (Table S2). The first is ‘*landscape connectivity*’, defined as the degree to which the landscape impedes or facilitates movement of individuals among habitat components^13^. Straight line distance (km), the distance to the nearest waterhole within the surrounding flood plain network^51^, is used here to quantify landscape connectivity (Table S2). Although landscape connectivity is representative of the physical relationship between habitat patches, it is static with respect to potential changes in connectivity driven by other processes. Thus, a second metric, ‘*hydrological connectivity*’ was quantified. It is defined as the extent to which temporal hydrology impedes or facilitates movement among habitat components; it captures the dynamic nature of ephemeral rivers and is representative of the boom and bust periods experienced by the Cooper Creek and associated fish communities. We quantify hydrological connectivity $(F)$ as standardised flow in the Cooper Creek network and time since last flood, $F=\,\mathrm{log}\,10(Q)+S$, where $Q$ is the Cooper Creek flow (both major flood and minor in channel flow events, Fig. 2a) and $S$ is time since last flood (Table S2). By using these two metrics, we encompass both the spatial and temporal connectivity environment; ‘*landscape connectivity*’ takes into account the differences in spatial habitat, whereas ‘*hydrological connectivity*’ encompasses the temporal connectivity component, calculated for the Cooper Creek river network.

### Model of Species Occurrence

**Figure 4:**
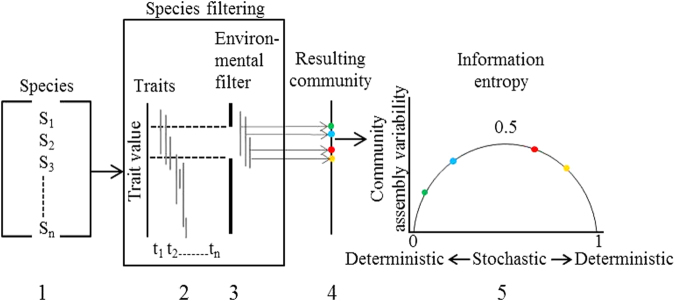
Species presence/absence data (1) and trait information (2) were used together with a GLMM to determine the presence/absence of each freshwater fish species. The final GLMM indicated that only few traits determined the probability of species prevalence (3). Predicted probabilities of presence/absence were input into a community assembly model that encompasses these trait values forming different resulting community assemblages (4). Each community represents differences in information entropy and community state, i.e. stochastic or deterministic (5).

Probability of occurrence of a species can be achieved directly from presence-absence data using logistic regression and related models. To identify which traits were most important in determining the presence/absence of freshwater fish species we used a generalised linear mixed effects model (GLMM) (Fig. 4). A principal components analysis (PCA) was initially performed to reduce multicollinearity among trait variables, reducing the initial 16 traits to 3. Fish species were ordinated with trait values that showed significant correlations in traits across species, explaining the maximum amount of variance (Fig. S1). The GLMM model predicted presence/absence of each fish species based on species’ traits (fixed effects) and random effects for waterhole and sampling time. The initial GLMM model comprised all (3) traits and two random effects. The trait variables included in the model were reduced using a step-wise (forward/backward elimination) procedure until maximum explanatory power was reached based on the lowest AIC (Akaike’s information criterion; Table S4)^57^. We also conducted stepwise simplification using likelihood ratios; the final model was the same for all methods.

To test for the putative role of connectivity as a predictor of species presence, we performed linear regressions between log odds (response variable) and either landscape or hydrological connectivity metrics. The log odds ratio quantifies the direction and magnitude of the outcome of interest,in this case the probability of species presence over the probability of species absence in a given waterhole at a particular time and the variable of interest (landscape and hydrological connectivity). All analyses were conducted using R (3.2.3) using the lme4 package^58^.

### Community Assembly Model

The predicted probabilities of species occurrences (presence/absence) at each time and waterhole, derived from the GLMM predictions were input into a community assembly model to calculate the probability of different community states, where a given state reflects a set of species’ presences and absences (Fig. 4). The probability of occurrence for each community state, *p*_*i*_, was calculated for every possible community as the joint probability of each species presence/absence for a given community state. There were 2^*n*^ possible community assemblages, where *n* is the number of species (*n* = 14).

The Shannon information entropy (*H*) for each time and waterhole was calculated:

1 $${H}_{j,k}=-\sum {p}_{i}\cdot {\mathrm{log}}_{2}({p}_{i})$$

where *j* is time, *k* is waterhole, *p*_*i*_ is the probability of occurrence of each community state.

Shannon information entropy is maximised at *n* species, which translates to equal probability of all community states, i.e. quantifying community assembly variability (Fig. 4). The key insight from the Shannon information entropy equation is that variation in assemblage structure will be maximised when $p(i)=0.5$ for all *i*. Thus the information entropy of re-assembly will increase for connectivity and trait variables that shift species towards $p(i)=0.5$ and decrease if the variables shift species away from $p(i)=0.5$ (Fig. 4). Shannon entropy has units of bits. Entropy of community states is maximised at the number of species in the community, so dividing entropy by the number of species would yield a relative measure. However here we use entropy on its natural scale, because its magnitude in bits is indicative of how variable the community could be relative to other communities.

### Trait Filtering

To investigate the effect of individual species traits on community entropy, a trait filtering procedure was employed. All significant terms of the fixed effects from the final GLMM model (specific trait values) were filtered sequentially following the GLMM fitting procedure (Table S5). For example, trait values for high dispersal ability were replaced with low dispersal ability and vice versa (Table S5). This trait replacement allowed us to test how specific combinations of trait values influence re-assembly. New predicted probabilities of species occurrences were generated following trait filtering and re-run in the community assembly model.

### Data Availability

For this study we complement a published dataset (data were originally collected and made available by Arthington *et al*.^51^, Balcombe and Arthington^30^).

## Electronic supplementary material

Supporting Information
