# Supplementary material for: Species traits and connectivity constrain stochastic community re-assembly
Source: Sci Rep. 2017 Oct 31;7:14424. doi: 10.1038/s41598-017-14774-2 (PMC5663852; doi:10.1038/s41598-017-14774-2)
Supplement: Supplementary file 1 — Supporting Information [file 41598_2017_14774_MOESM1_ESM.pdf]

Supporting Information

Species traits and connectivity constrain stochastic community re-assembly

Rebecca E. Holt, Christopher J. Brown, Thomas A. Schlacher, Fran Sheldon, Stephen R. Balcombe and Rod M. Connolly.

Table S1. Fish species found in waterholes included within this study of the Cooper Creek, and number of waterholes occupied by those species in the 8 sampling times from April 01 to December 2004.

Species	Common Name	No. of waterholes occupied (max = 4)							
		Apr 01	Sept 01	Oct 02	May 03	Mar 04	Jun 04	Oct 04	Dec 04
Ambassis sp.	Northwest ambassis	3	2	2	1	1	1	2	2
Bidyanus welcii	Welch's grunter (Silver bream)	2	0	0	1	3	2	0	1
Carassius auratus	Goldfish	3	4	2	0	0	0	1	0
Gambusia holbrooki	Mosquitofish	0	0	0	2	1	0	0	0
Hypseleotris sp.	Carp gudgeons	2	0	1	2	1	2	2	3
Leiopetherapon unicolor	Spangled perch	4	3	4	1	4	4	4	3
Macquaria sp.	Lake Eyre golden perch	4	3	3	4	4	4	4	3
Melanotaenia splendida subsp. *Tatei*	Desert rainbowfish	4	3	3	4	4	4	3	2
Nematalosa erebi	Bony bream	3	2	3	3	3	3	3	2
Neosiluroides cooperensis	Cooper Creek tandan	2	0	2	0	3	2	1	1
Neosilurus hyrtlii	Hyrtl's tandan	4	4	4	2	4	4	4	3
Porochilus argenteus	Silver tandan	4	4	4	4	4	4	4	3
Retropinna semoni	Australian smelt	2	2	2	4	2	2	4	2
Scortum barcoo	Barcoo grunter	4	2	0	2	4	3	2	1

Table S2. Physical and hydrological characteristics measured at Cooper Creek.

Floodplain characteristics	Acronym	Units	Description
River flow at time of sampling	RF	ML/day	Cooper creek flow and flood data, Bureau of Meterology.
Straight line distance to nearest waterhole	SLD*	km	Distance to the nearest waterhole within the surrounding flood plain network (Arthington *et al*. 2005).
Time since last flood	TSLF	Months	Period of drought in area of study since last flood

*****Quantified during April 2000 original sampling trip (See Arthington *et al*. 2005), SLD was used as the metric for landscape connectivity, describing the proximity to the nearest waterhole surrounding the floodplain network.

Table S3. Species traits quantified for 14 freshwater fish species.

Fish Species Trait	Description
Age at maturity (months)	Mean age at maturation
Body size (cm)	Standard length*
Conductivity ($\mu S.cm^{-1}$)	Conductivity tolerance
Dispersal category	Defines a species vagility
Dissolved oxygen tolerance (O_2 mg L^{-3})	Dissolved oxygen tolerance
Egg size (mm)	Mean diameter of mature (fully yolked) ovarian oocyte
Fecundity	Total fecundity, no. of eggs or offspring per breading season
Length at maturity (cm)	Mean length at maturation
Longevity (years)	Maximum potential lifespan
Maximum length (cm)	Maximum body length
Parental Care	Category representing whether they perform parental care or not
pH	pH tolerance
Spawning category	Single spawning per season, batch / repeat / protracted spawner per season
Temperature (°C)	Temperature tolerance
Time to hatch (days)	Time to egg hatch.
Turbidity (NTU)	Turbidity tolerance

*Standard length is measured from the tip of the snout to the posterior end of the last vertebra, excluding the length of the caudal fin.

Where trait information was unknown for lesser known species, proxy species that are ecologically similar were used; *Tandanus tandanus* was used for *Neosiluroides cooperensis* and *Scortum parviceps* for *Scortum barcoo* (S. Balcombe, pers.comm.). Ecologically similar species in this case are defined as a phylogenetically bounded group of species that use a similar set of resources within a community (Fauth *et al*. 1996).

Table. S4. Summary of generalised linear mixed effect model (GLMM) comparisons using Akaike's Information Criterion (AIC).

Model Structure	AIC
Dissolved oxygen tolerance + fecundity + dispersal category	522.2*
Dissolved oxygen tolerance + fecundity	549.1
Dissolved oxygen tolerance	561.0
Dissolved oxygen tolerance + fecundity + dispersal category + pH	587.6
Dissolved oxygen tolerance + fecundity + dispersal category + conductivity	588.4
Dissolved oxygen tolerance + fecundity + dispersal category + turbidity	574.0
Dissolved oxygen tolerance + fecundity + dispersal category + egg size	584.0
Dissolved oxygen tolerance + fecundity + dispersal category + age at maturity	576.2
Dissolved oxygen tolerance + fecundity + dispersal category + body size	580.9
Dissolved oxygen tolerance + fecundity + dispersal category + length at maturity	602.5
Dissolved oxygen tolerance + fecundity + dispersal category + longevity	649.8
Dissolved oxygen tolerance + fecundity + dispersal category + maximum length	656.3
Dissolved oxygen tolerance + fecundity + dispersal category + parental care	684.9
Dissolved oxygen tolerance + fecundity + dispersal category + spawning category	647.2
Dissolved oxygen tolerance + fecundity + dispersal category + temperature	616.5
Dissolved oxygen tolerance + fecundity + dispersal category + time to hatch	678.0

*Final model structure

Table. S5. Trait manipulation matrix

Trait manipulation scenario	Original value	Manipulated value
Low tolerance to low dissolved oxygen	3	1
High tolerance to low dissolved oxygen	1	3
High dispersal ability	1	3
Low dispersal ability	3	1
Low total fecundity	3, 4, 5	1
High total fecundity	1, 2, 3	4, 5

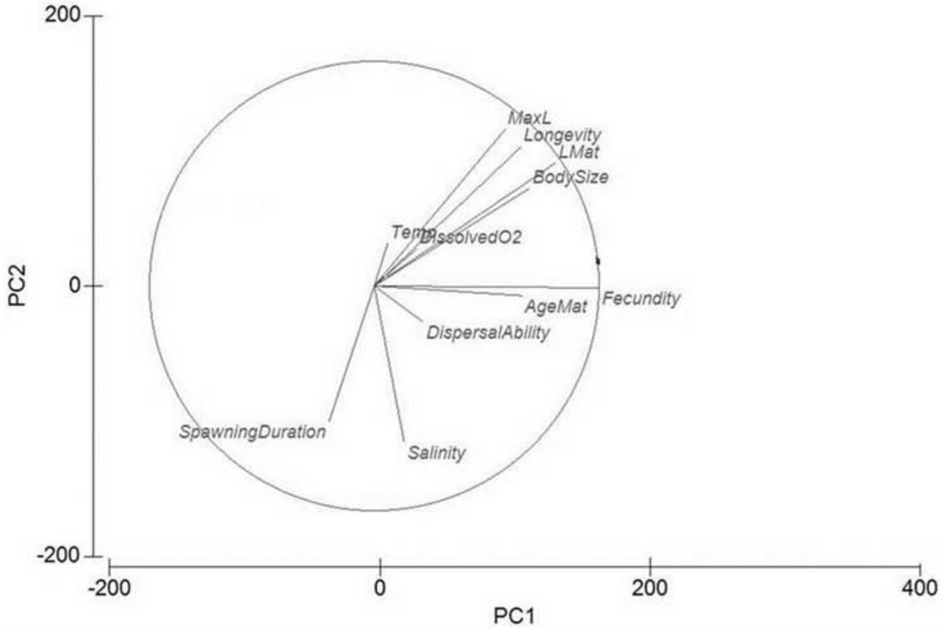

Figure S1. PCA analysis showing the distribution of species traits and fish species included in analyses. PC1, 97.3 % variation. PC2, 98.7% % variation.
